# Conduction system pacing vs. biventricular pacing for cardiac resynchronization: the CSP-SYNC randomized single centre study

**DOI:** 10.1093/europace/euaf192

**Published:** 2025-08-23

**Authors:** David Žižek, Tadej Žlahtič, Miha Mrak, Maja Ivanovski, Jernej Štublar, Dinko Zavrl Džananović, Jakob Peterlin, Marta Cvijić, Anja Zupan Mežnar

**Affiliations:** Cardiology Department, University Medical Centre Ljubljana, Zaloška 2, 1000 Ljubljana, Slovenia; Faculty of Medicine, University of Ljubljana, Vrazov trg 2, 1000 Ljubljana, Slovenia; Cardiology Department, University Medical Centre Ljubljana, Zaloška 2, 1000 Ljubljana, Slovenia; Faculty of Medicine, University of Ljubljana, Vrazov trg 2, 1000 Ljubljana, Slovenia; Cardiology Department, University Medical Centre Ljubljana, Zaloška 2, 1000 Ljubljana, Slovenia; Cardiology Department, University Medical Centre Ljubljana, Zaloška 2, 1000 Ljubljana, Slovenia; Faculty of Medicine, University of Ljubljana, Vrazov trg 2, 1000 Ljubljana, Slovenia; Cardiology Department, University Medical Centre Ljubljana, Zaloška 2, 1000 Ljubljana, Slovenia; Cardiology Department, University Medical Centre Ljubljana, Zaloška 2, 1000 Ljubljana, Slovenia; Faculty of Medicine, University of Ljubljana, Vrazov trg 2, 1000 Ljubljana, Slovenia; Cardiology Department, University Medical Centre Ljubljana, Zaloška 2, 1000 Ljubljana, Slovenia; Faculty of Medicine, University of Ljubljana, Vrazov trg 2, 1000 Ljubljana, Slovenia; Cardiology Department, University Medical Centre Ljubljana, Zaloška 2, 1000 Ljubljana, Slovenia; Faculty of Medicine, University of Ljubljana, Vrazov trg 2, 1000 Ljubljana, Slovenia

**Keywords:** Cardiac resynchronization therapy, Biventricular pacing, Left bundle branch area pacing, Left bundle branch block, Conduction system pacing

## Abstract

**Aims:**

There are limited prospective randomized studies comparing left bundle branch area pacing (LBBAP) and biventricular (BiV) pacing for cardiac resynchronization therapy (CRT). The study tested whether LBBAP is non-inferior to BiV pacing in patients with Class I indication for CRT.

**Methods and results:**

The CSP-SYNC study is an investigator-initiated, randomized, single-centre study. Sixty-two patients were randomized 1:1 to LBBAP or BiV. The primary study endpoint was the change in left ventricular ejection fraction (LVEF) at 6 months. Secondary endpoints included changes in echo and clinical parameters after 6 months and 12 months. Thirty-one patients were randomized to each arm. Most patients were males (71%), and 32% had ischaemic cardiomyopathy. At 6 months, similar improvement of LVEF was observed in the LBBAP group compared to the BiV group [14.0% (95% confidence interval (CI): 11.2–16.8) in LBBAP vs. 8.5% (95% CI: 5.6–11.2) in BiV] with a mean intergroup difference of 5.6% (95% CI: 1.6–9.5; *P* < 0.001 for non-inferiority). Both groups showed comparable decrease in LVESV [−64 mL (95% CI: −78 to −50) vs. −40 mL (95% CI: −54 to −25) respectively, mean difference −24 mL (CI 95%: −44 to −4); *P* < 0.001 for non-inferiority] and changes in 6-min walk test (*P* < 0.001 for non-inferiority) and NYHA class (*P* = 0.011 for non-inferiority). Temporal trends of LV remodelling and heart failure hospitalization rates were also comparable.

**Conclusion:**

In patients with a Class I indication for CRT, LBBAP was non-inferior to BiV pacing in improving LVEF and provided similar structural and electrical remodelling.

## Graphical Abstract

**Graphical Abstract:**
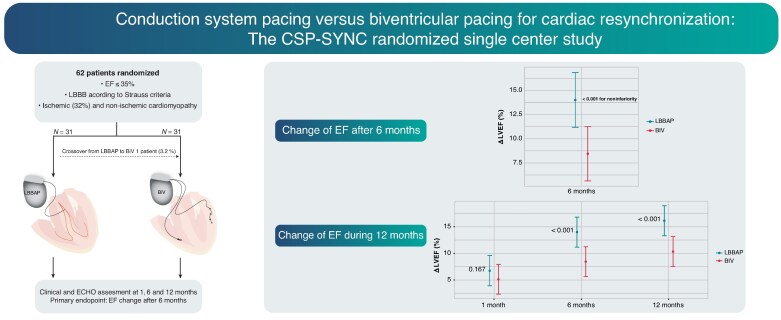

What’s new?

- Randomized data comparing left bundle branch area pacing (LBBAP) and biventricular pacing for resynchronization is scarce. This is a randomized trial addressing both pacing modalities in patients with a Class I indication for resynchronization therapy regardless of heart failure aetiology.
- The change in left ventricular ejection fraction after LBBAP was non-inferior to biventricular pacing at the preplanned 6-month follow-up.
- Left bundle area pacing exhibited similar electrical and structural reverse remodelling after 12 months.
- Patients receiving LBBAP had significantly higher rates of ejection fraction normalization and a trend towards lower heart failure hospitalizations.

## Introduction

Current guidelines for cardiac resynchronization therapy (CRT) provide a Class I recommendation for biventricular (BiV) pacing in symptomatic heart failure (HF) patients with sinus rhythm, left ventricular ejection fraction (LVEF) ≤ 35%, QRS duration ≥150 ms, and left bundle branch block (LBBB) QRS morphology.^1^ Despite a clear impact on survival,^2,3^ several limitations like unfavourable venous anatomy, phrenic nerve stimulation, high pacing thresholds, and altered myocardial depolarization may limit adequate resynchronization of BiV pacing.^4^ Conduction system pacing (CSP) utilizing either His bundle pacing or left bundle branch area pacing (LBBAP) can bypass the pathological region in the cardiac conduction system and result in a nearly physiological activation pattern in patients with LBBB.^5^

While some randomized data have shown that His bundle pacing can improve cardiac function in HF patients with LBBB, clear advantages over BiV pacing were hampered by higher pacing thresholds and significant crossover rates.^6,7^ On the other hand, several observational studies have demonstrated that LBBAP offers advantages over BiV pacing beyond effective resynchronization, including shorter procedural and fluoroscopy times, fewer lead-related complications, lower pacing thresholds, and high implantation success rates.^8–12^ However, prospective randomized studies comparing standard BiV pacing and LBBAP are limited.^13–15^

Our study evaluated whether LBBAP is non-inferior to BiV pacing in improving LVEF at the 6-month follow-up in patients with a Class I indication for CRT. Temporal echocardiographic and electrocardiographic changes over 12 months were also assessed as secondary endpoints.

## Methods

### Study design

The CSP-SYNC (Conduction System Pacing Versus Biventricular Pacing for Cardiac Resynchronization) is an investigator-initiated, randomized, non-inferiority study conducted at the University Medical Centre Ljubljana, Slovenia, between January 2022 and October 2024. The study is in compliance with the Declaration of Helsinki and was approved by the Medical Ethics Committee of the Republic of Slovenia (Approval Number: 0120–227/2021/8). All patients provided written informed consent. All data were registered and securely stored in a REDCap database. The executive committee designed and conducted the trial, which was registered in ClinicalTrials.gov (NCT05155865).

### Study population and sample size

Consecutive patients referred for CRT indication were evaluated and enrolled in the study if they met the inclusion criteria. The inclusion criteria were defined according to Class I recommendation of the current CRT guidelines: age ≥ 18 years, symptomatic HF despite optimal medical therapy, sinus rhythm, LVEF ≤ 35%, QRS duration ≥ 150 ms, and LBBB QRS morphology. Left bundle branch block was defined using the Strauss criteria.^16^ Exclusion criteria included previous mechanical tricuspid valve replacement, unstable angina, acute myocardial infarction or coronary revascularization within the past 6 months, persistent or permanent atrial fibrillation, high-degree atrioventricular block, life expectancy <12 months, and pregnancy or breastfeeding.

Calculations of sample size were performed using the G*Power calculator.^17^ Based on the findings of a previous study,^8^ we expected an absolute increase in LVEF from baseline to 6 months of 18% in the LBBAP group and 13% in the BiV group, with a standard deviation (SD) of 5%. Assuming a parallel-group design with equal allocation, and accounting for an expected crossover of 15% in the LBBAP and 10% in the BiV, we calculated that 48 participants would yield 90% power to detect the intergroup difference using a one-sided, two-sample *t*-test, with a non-inferiority margin of 2.5 at a significance level of 0.01 (adjusted from 0.05 to account for multiple comparisons). Allowing for a 10% loss to follow-up rate, the sample size was determined to be 31 participants for each group.

### Randomization

Eligible patients were randomly assigned in a 1:1 ratio to LBBAP or BiV pacing. To ensure baseline comparability in key predictors of CRT response, randomization was stratified by age groups (per decade), LVEF (<25% vs. ≥25%), and QRS width (<150 ms vs. ≥150 ms). A real-time electronic data capture system (REDCap) was utilized for data collection and randomization. Patients were blinded to treatment allocation in a single-blind design.

### Study endpoints

The primary study endpoint was the change in LVEF at the 6-month follow-up between the two groups. Secondary endpoints included temporal changes at 1, 6, and 12 months in LVEF, left ventricular end-systolic volume (LVESV), and paced QRS duration. Secondary endpoints related to laboratory parameters and symptomatic improvement included changes in N-terminal pro-B-type natriuretic peptide levels, 6-min walk test distance, New York Heart Association (NYHA) class, and EuroQol visual analogue scale score after 6 months.

We also evaluated echocardiographic improvement at 6 months stratified according to echocardiographic response and super-response. Echocardiographic response was defined as a reduction of LVESV ≥ 15%, and echocardiographic super-response was defined as normalization of EF (LVEF ≥ 50%) after resynchronization.

Further clinical endpoints included the composite of cardiovascular death or worsening HF, as well as its individual components. A worsening HF event was defined as hospitalization due to HF or unplanned emergency department visit requiring intravenous diuretic therapy. Procedural complications and adverse events during follow-up were systematically recorded.

### Procedures

All devices were implanted according to standard clinical practice. Three experienced operators performed all device implantations (D.Ž., A.Z.M., and I.Z.). The physician had the discretion to select the manufacturer and type of device. In the BiV group, either a pacing device (CRT-P) or a defibrillator (CRT-D) was used, while in the LBBAP group, a dual-chamber pacemaker, dual-chamber implantable cardioverter defibrillator (ICD) with DF-1 connector, and CRT-D device (with DF-1/IS-1 or DF-4/IS-1 connectors) were possible.

In BiV devices, the right ventricular (RV) lead was positioned in the RV apex or septum. The procedure was considered successful if predefined criteria were met, including non-apical posterolateral or lateral LV lead placement (when permitted by the venous anatomy), an LV pacing threshold < 3 V at a pulse width of 0.5 ms, and a phrenic nerve stimulation threshold at least 1 V higher than the LV pacing threshold. Commercially available devices and leads were used. Crossover to LBBAP was allowed if the LV lead could not be positioned due to anatomical constraints, phrenic stimulation, or high pacing thresholds.

The procedural steps for delivering LBBAP were previously reported.^8–10^ In brief, LBBAP was attempted with two different combinations of pacing leads and delivery sheaths. The Medtronic 4.1 Fr bipolar active fixation lead (SelectSecure 3830, Medtronic, Minneapolis, MN, USA) was delivered through a long pre-shaped sheath (C315 His, Medtronic, Minneapolis, MN, USA). Alternatively, implantations were performed with a 5.6 Fr stylet-driven pacing lead (Solia S60, Biotronik, Berlin, Germany) delivered through a pre-shaped sheath (Selectra 3D, Biotronik, Berlin, Germany). Electrophysiological system Bard LabSystem Pro (Boston Scientific, Lowell, MA, USA) or EP-TRACER 2 Portable (CardioTek B.V., Sittard, the Netherlands) were used to record and analyse intracardiac electrograms and surface electrocardiogram (ECG) at a sweep speed of 100 mm/s. The LBBAP lead deployment site was determined over a wider mid-septal area using fluoroscopic delineation of the tricuspid ring, the paced QRS morphology (‘W’ pattern in lead V1, polarity discordance in leads II and III), and endocardial electrograms. Lead depth in the interventricular septum during implantation was monitored using progressive changes in paced QRS morphology, the presence of fixation beats, local endocardial electrogram, impedance, and current of injury. The number of lead implantation attempts, as well as the final position, were at the discretion of the implanter. To accept the LBBAP lead position, deep septal lead placement had to be achieved with unipolar paced Qr or qR morphology in lead V1 along with at least one of the following criteria based on previous publications: (i) diagnostic QRS morphology transition during threshold testing (a sudden change from the initial QRS morphology pattern to either selective LBBAP or LV septal pacing); (ii) pacing stimulus to peak R-wave time in lead V6 ≤ 90 ms; (iii) V6-V1 interpeak interval > 44 ms; and (iv) diagnostic QRS morphology transition during programmed stimulation. Crossover to BiV was allowed in cases of failed LBBAP due to high threshold, inability to penetrate the septum, or failure to meet predefined LBBAP criteria. Fluoroscopy time and total procedural duration of device implantation were also collected and compared in both groups.

### Programming

Echocardiography- and ECG-based atrioventricular and interventricular delay optimization of the BiV devices was performed after implant to ensure LV capture (Q wave in lead I, R wave in lead V1) and optimal QRS duration. Multipoint pacing was not used. In patients with LBBP, the ECG-based atrioventricular interval was optimized with the aim of achieving the shortest QRS interval by allowing fusion with intrinsic right bundle ventricular activation. Pacing outputs were programmed to ensure stable capture of the conduction system or BIV capture.

### Follow-up

Before discharge, ECG, X-ray, and device interrogation were performed. Clinical visits were scheduled at 1, 6, and 12 months after device implantation and every 6 months thereafter. Patients were followed until the study ended and for at least 12 months. Each visit consisted of a physical examination, device interrogation, and evaluation of ECG, guideline-directed medical therapy, history of hospital admissions and unplanned visits, and arrhythmia episodes.

### Echocardiographic and electrocardiographic measurements

Echocardiography examinations and ECG recordings were performed at baseline, 1, 6, and 12 months. Echocardiograms (Vivid E95, Vingmed-General Electric, Horten, Norway) were performed by two operators (M.C., D.Z.D.) blinded to treatment allocation. Left ventricular end-diastolic volume, LVESV, and LVEF were calculated by the biplane Simpson method of discs. The severity of mitral (MR) and tricuspid regurgitation (TR) was graded using a multiparametric integrative approach and was subclassified into three grades (mild/moderate/severe).

The paced QRS duration was measured from the beginning of the pacing stimulus to the end of the QRS complex on 12-lead ECGs at a sweep speed of 100 mm/s using digital callipers (Cardiax ECG, IMED Kft, Budapest, Hungary). Three cardiologists (T.Ž., M.M., A.Z.M.), unaware of the study endpoint, independently measured QRS duration, and the average of 3 measurements was calculated. In the event that interobserver discrepancy exceeded 10 ms, the measurements were reviewed until consensus was reached.

### Statistical analysis

Categorical variables are presented as frequencies and percentages; continuous variables as mean ± SD or median [interquartile range (IQR)], depending on normality by the Kolmogorov–Smirnov test. Baseline and procedural differences were assessed using Student’s *t*-test or Wilcoxon rank-sum for continuous variables, and *χ*^2^ or Fisher’s exact tests for categorical variables. Time-to-event outcomes were compared by the log-rank test and Cox proportional hazards model. Analysis was performed under an intention-to-treat framework.

Longitudinal LVEF, LVESV, and QRS data were analysed with linear mixed models (random intercepts, time as a categorical factor), reporting BiV vs. LBBAP contrasts at 1, 6, and 12 months. N-terminal pro-B-type natriuretic peptide post–pre changes were tested by Mann–Whitney; 6-min walk test and EuroQol visual analogue scale score by *t*-test; and NYHA class by ordinal regression. The difference in grades of mitral and TR between the baseline and the 12-month follow-up was tested using ‘The Sign’ test.

For all endpoints, we performed one-sided non-inferiority tests and two-sided superiority tests at significance level *α* = 0.05, with Holm’s correction across 13 independent tests (ordering comparisons by non-inferiority *P*-values). The non-inferiority margin for the primary endpoint was set at a 2.5% absolute difference in LVEF. This margin was considered clinically non-relevant in terms of its potential impact on clinical outcomes and is supported by prior publications.^14,15,18^ Secondary endpoint non-inferiority margins were similarly considered as not clinically relevant and based on previous data: 12 ms for QRS narrowing, 15 mL for ΔLVESV, 1 for NYHA class change, 50 m for 6-min walk test, 10 points for EuroQol visual analogue scale score, and 100 ng/L for N-terminal pro-B-type natriuretic peptide levels.^14^  ^,15,18^ Analyses without correction for multiple analysis (unadjusted) were also performed and are presented in the Supplementary material (Supplementary material online, *Table S1*). Analyses were done in R version 4.4.2.

## Results

### Patient population

**Figure 1:**
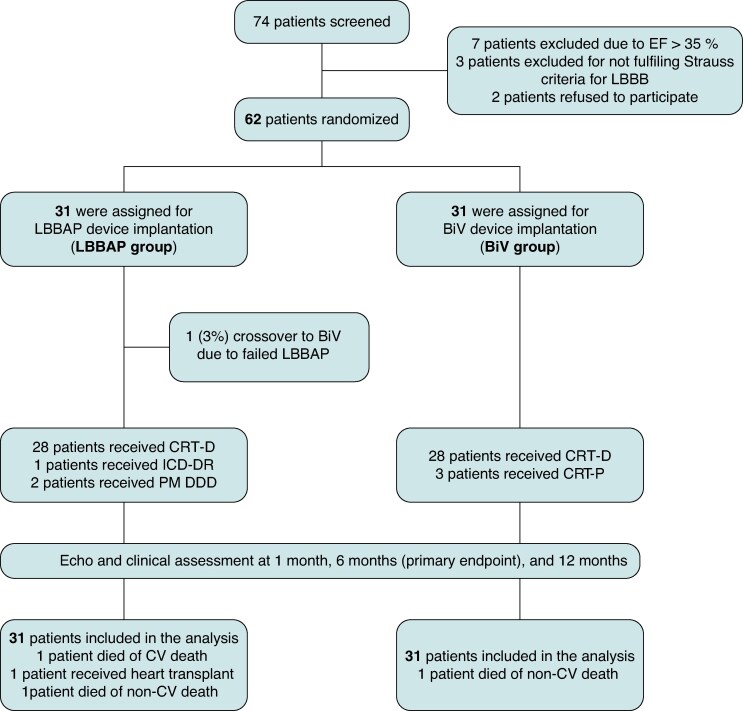
Study flowchart. Randomization and allocation of patients are shown. BiV, biventricular pacing; CRT-D, cardiac resynchronization therapy device with cardioverter defibrillator; CRT-P, cardiac resynchronization therapy device; ECHO, echocardiography; EF, ejection fraction; LBBB, left bundle branch block; LBBAP, left bundle branch area pacing; PM DDD, dual-chamber pacemaker; ICD-DR, dual-chamber implantable cardioverter defibrillator; CV, cardiovascular.

A total of 74 patients were screened for inclusion. Seven were excluded due to LVEF > 35%, three due to LBBB not meeting Strauss criteria, and two declined participations. Finally, 31 patients were randomized to the LBBAP group and 31 to the BiV group (*Figure 1*).

Baseline characteristics were comparable between both groups and are shown in *Table 1*. The median age was 67 years (IQR 59–73), 71% of patients were male, and 32% had ischaemic cardiomyopathy. The mean baseline LVEF was 29 ± 6%, and the mean baseline QRS duration was 174 ± 18 ms. Most patients (72%) were in NYHA functional class II. Over 90% of the patients were receiving all four guideline-recommended HF medications. No patients were lost to follow-up.

**Table 1:** Baseline patient characteristics.

	LBBAP (*n* = 31)	BiV (*n* = 31)
Male, *n* (%)	23 (74%)	21 (67%)
Age, median (IQR) years	65 (59–72)	70 (57–77)
Ischaemic cardiomyopathy, *n* (%)	11 (35%)	9 (29%)
Glomerular filtration, median (IQR) mL/min	79 (61–90)	71 (45–88)
BMI, median (IQR) kg/m**^2^**	28 (25–32)	27 (24–34)
NYHA functional class, *n* (%)		
II	24 (77%)	21 (68%)
III	7 (23%)	10 (32%)
EQ VAS score, median (IQR) %	65 (50–80)	70 (50–80)
QRS, ±SD ms	176 ± 18	170 ± 19
LVEF, ±SD %	30 ± 5	28 ± 6
LV end-diastolic volume, ±SD mL	255 ± 81	257 ± 64
LV end-systolic volume, ±SD mL	179 ± 65	188 ± 57
Medical treatment		
Beta-blockers, *n* (%)	31 (100%)	30 (97%)
ACEI/ARB, *n* (%)	2 (6%)	0 (0%)
ARNI, *n* (%)	29 (94%)	30 (97%)
Aldosterone antagonist, *n* (%)	31 (100%)	30 (97%)
SGLT-2 inhibitors, *n* (%)	29 (94%)	29 (94%)
Loop diuretics, *n* (%)	9 (29%)	11 (35%)

ACEI, angiotensin-converting enzyme inhibitors; ARB, angiotensin II receptor blockers; ARNI, angiotensin receptor neprilysin inhibitors; BiV, biventricular pacing; BMI, body mass index; EQ VAS score, EuroQol visual analogue scale score; LBBAP, left bundle branch area pacing; LV, left ventricular; LVEF, left ventricular ejection fraction; IQR, interquartile range; NYHA, New York Heart Association; SD, standard deviation, SGLT-2, sodium–glucose cotransporter-2.

### Primary endpoint

**Figure 2:**
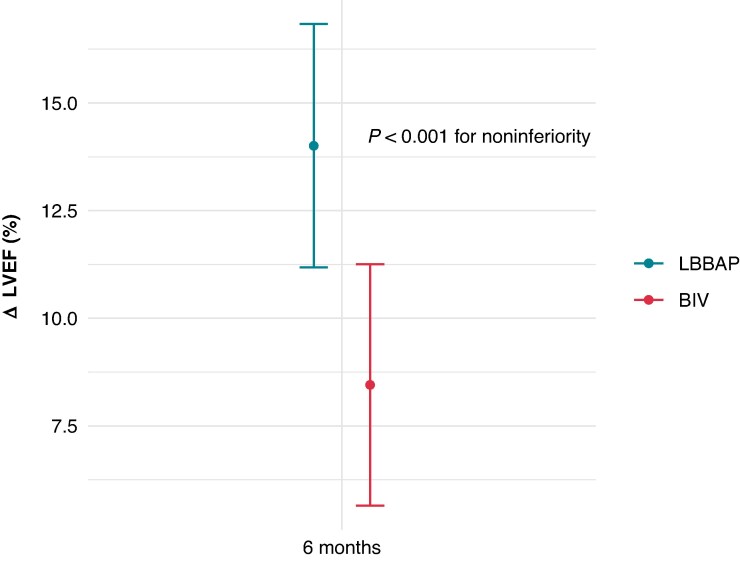
Primary outcome. Change in left ventricular ejection fraction after 6 months of resynchronization with LBBAP and BiV. Marked values represent the mean difference with 95% confidence intervals. Result of the longitudinal test with non-inferiority *P*-value corrected for multiple analysis with Holm’s method is provided. BiV, biventricular; LBBAP, left bundle branch area pacing; LVEF, left ventricular ejection fraction; ΔLVEF = (LVEF at 6 months − baseline LVEF).

**Figure 3:**
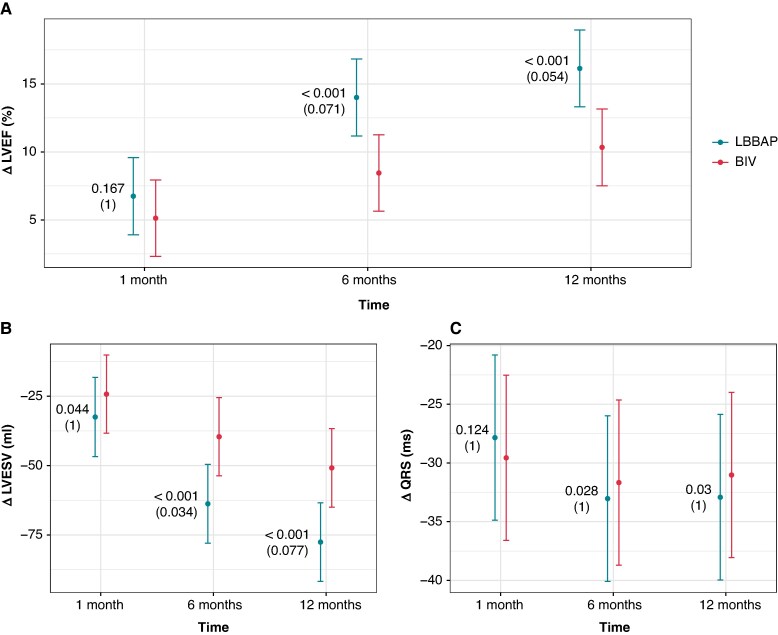
Structural and electrical remodelling during 12 months. Comparison of LVEF (*A*), LVESV (*B*), and QRS duration (*C*) differences between baseline and 1, 6, or 12 months of therapy in LBBAP and BiV arm. Marked values represent mean difference with 95% confidence intervals. Results of the longitudinal tests with non-inferiority *P*-values and superiority *P*-values in parentheses corrected for multiple analysis with Holm’s method are provided on graphs for individual month. LVESV, left ventricular end-systolic volume, Δ difference between baseline and 1, 6, or 12 months of resynchronization therapy. Other abbreviations as in *Figure 2*.

At 6-month follow-up, LVEF improved by 14.0% (95% CI: 11.2–16.8) in the LBBAP group and by 8.5% (95% CI: 5.6–11.2) in the BiV group, with a mean intergroup difference of 5.6% (95% CI: 1.6–9.5; *P* < 0.001 for non-inferiority) (*Table 2* and *Figure 2*). Time trend analysis of LVEF change during the 12-month period showed a similar intergroup difference [mean difference 5.8% (95% CI: 1.8–9.8); *P* < 0.001 for non-inferiority]. However, 1 month after implantation, the difference in LVEF improvement did not reach statistical significance (*P* = 0.167 for non-inferiority) (*Figure 3*).

**Table 2:** Echocardiographic and electrocardiographic endpoints after 6 months with results of the longitudinal tests with non-inferiority *P*-values and superiority *P*-values. *P*-values are adjusted with Holm’s method, and 95% confidence intervals are unadjusted.

	LBBAP	BiV	Mean difference between groups (95% CI)	*P*-value non-inferiority	*P*-value superiority
	Difference (95% CI)	Difference (95% CI)			
Primary					
ΔLVEF (%)	14.0 (11.2–16.8)	8.5 (5.6–11.2)	5.6 (1.6–9.5)	< 0.001	0.071
Secondary					
ΔLVESV (mL)	−64 (−78 to −50)	−40 (−54 to −25)	−24 (−44 to −4)	< 0.001	0.034
ΔQRS (ms)	−33 (−40 to −26)	−32 (−39 to −25)	−1 (−11 to 9)	0.028	1

LVEF, left ventricular ejection fraction; LVESV, left ventricular end-systolic volume.

As the primary endpoint was met, we also performed an exploratory analysis testing for superiority, which was non-significant at both 6 and 12 months (*P* = 0.071 and *P* = 0.054, respectively) (*Table 3*).

**Table 3:** Time trends of echocardiographic and electrocardiographic measurements with results of the longitudinal tests with non-inferiority *P*-values and superiority *P*-values in parentheses. *P*-values are adjusted with Holm’s method, and 95% confidence intervals are unadjusted.

	Mean difference between groups (95% CI)	*P*-value	Mean difference between groups (95% CI)	*P*-value	Mean difference between groups (95% CI)	*P*-value
	Baseline to 1 month		Baseline to 6 months		Baseline to 12 months	
Δ LVEF (%)	1.6 (−2.4 to 5.6)	0.167 (1)	5.6 (1.6 to 9.5)	< 0.001 (0.071)	5.8 (1.8 to 9.8)	< 0.001 (0.054)
Δ LVESV (mL)	−8 (−28 to 12)	0.044 (1)	−24 (−44 to −4)	< 0.001 (0.034)	−27 (−47 to −7)	< 0.001 (0.077)
Δ QRS (ms)	2 (−8 to 12)	0.124 (1)	−1 (−11 to 9)	0.028 (1)	−2 (−12 to 8)	0.03 (1)

LVEF, left ventricular ejection fraction; LVESV, left ventricular end-systolic volume.

### Secondary endpoints

At 6-month follow-up, LVESV decreased by −64 mL (95% CI: −78 to −50) in the LBBAP group and by −40 mL (95% CI: −54 to −25) in the BiV group. The decrease in LVESV was comparable between both groups with a mean intergroup difference of −24 mL (95% CI: −44 to −4; *P* < 0.001 for non-inferiority) (*Table 2*). Non-inferiority for LVESV change was also demonstrated at both 1 and 12 months (*P* = 0.044 and *P* < 0.001, respectively). Additional analysis for the superiority of LVESV change showed greater volume decrease in the LBBAP group after 6 months (*P* = 0.034) (*Figure 3* and *Table 3*).

At 6-month follow-up, the QRS duration decreased by −33 ms (95% CI: −40 to −26) in the LBBAP group and by −32 ms (95% CI: −39 to −25) in the BiV group. Non-inferiority was reached at 6 and 12 months (*P* = 0.028 and *P* = 0.03, respectively), but not after the first month (*P* = 0.124). Superiority analysis was not significant (*Figure 3*).

After 6 months, N-terminal pro-B-type natriuretic peptide decreased from 747 ng/L (IQR 382–1452) to 265 ng/L (IQR 97–627) in the LBBAP group and from 1129 ng/L (IQR 424–3534) to 562 ng/L (IQR 235–1764) in the BiV group. The difference in N-terminal pro-B-type natriuretic peptide reduction after 6 months did not reach the non-inferiority threshold (*P* = 0.365).

The 6-min walk test distance improved from 405 m (IQR 330–504) to 450 m (IQR 374–510) in the LBBAP group and from 408 m (IQR 258–484) to 420 m (IQR 295–480) in the BiV group, showing non-inferiority between groups (*P* < 0.001).

At 6-month follow-up, 65% of the LBBAP patients improved by at least one NYHA class, which was comparable to 52% of patients in the BiV group (*P* = 0.011 for non-inferiority). Median EuroQol visual analogue scale score improved from 65 (IQR 50–80) to 80 (IQR 60–90) in the LBBAP group and from 70 (IQR 50–80) to 80 (IQR 60–90) in the BiV group. The intergroup difference did not meet the threshold for non-inferiority (*P* = 0.366) (*Table 4*).

**Table 4:** NT-proBNP level and symptomatic change after 6 months of therapy with results for non-longitudinal tests with *P*-values for non-inferiority tests and *P*-values for superiority tests. All *P*-values are adjusted with Holm’s method.

	*P*-value non-inferiority	*P*-value superiority
NT-proBNP	0.365	0.889
6-MWT	< 0.001	1
EQ VAS score	0.366	1
NYHA functional class	0.011	1

EQ VAS score, EuroQol visual analogue scale score; NYHA, New York Heart Association; NT-proBNP, N-terminal pro-B-type natriuretic peptide; 6-MWT, 6-min walk test.

### Echocardiographic response and clinical events

The predefined echocardiographic response was observed in 83% of patients in the LBBAP group and 61% in the BiV group (*P* = 0.09). The predefined super-response was more common in the LBBAP group. There were 40% super-responders in the LBBAP group and only 3% in the BiV group (*P* < 0.01).

There was no intergroup difference in the percentage of ventricular pacing at 6 months (99% vs. 98%, *P* = 0.10).

Over a mean follow-up of 22.1 ± 7.5 months, the composite endpoint of cardiovascular death or worsening of HF occurred in two (6.5%) patients in the LBBAP group and in seven (22.6%) patients in the BiV group (hazard ratio 0.26; 95% CI: 0.06–1.27; *P* = 0.10). There were two (6.5%) HF hospitalizations in the LBBAP group and seven (22.6%) in the BiV group (hazard ratio 0.26; 95% CI: 0.05–1.26; *P* = 0.09). One cardiovascular death and one heart transplant occurred in the LBBAP group, while there was one non-cardiovascular death reported in the BiV group.

### Procedural data, valvular regurgitation, and complications

In the LBBAP group, there was one crossover (3.2%) to BiV due to failure of LBB capture despite several lead positioning attempts. While narrowing of paced QRS was observed in all remaining procedures in the LBBAP arm, LBBAP capture was confirmed in 28 out of 30 patients (93%). Selective LBBAP capture was observed during intraoperative unipolar measurements in 22 out of 28 patients (79%) (Supplementary material online, *Table S3*). There were no crossovers in the BiV group. Coronary sinus lead was placed in the anterolateral vein in 4 (13%), lateral vein in 14 (45%), and posterolateral vein in 13 (42%) patients.

In the LBBAP group, 28 patients received CRT-defibrillator devices, 1 patient received a dual-chamber ICD DF-1 device, and 2 received dual-chamber pacemakers. In the BiV group, 28 patients received CRT with a defibrillator, and 3 patients were implanted with CRT pacing only devices.

Procedural duration time in the LBBAP group was comparable with BiV (70 min (IQR 60–85) vs. 74 min (IQR 59–90), *P* = 0.40). In contrast to the procedural time, there was shorter fluoroscopy time in the LBBAP group [9 min (IQR 6–13) vs. 13 min (IQR 10–21), *P* < 0.01]. Pacing thresholds for LBBAP and LV lead capture did not differ (*P* = 0.06), while pulse width was shorter in the BiV arm (*P* < 0.01).

In our study cohort, none of the patients had severe MR or TR at the inclusion, and only 4 patients had moderate MR before device implantation. In the LBBAP arm, there were no significant changes in the severity of MR (*P* = 1.00) or TR (*P* = 0.581) between baseline and 12-month follow-up. Similarly, the grade of MR severity remained stable between the baseline and the 12-month follow-up in the BiV arm (*P* = 0.092), while the grade of TR severity was higher at the 12-month follow-up than at baseline (*P* = 0.004). These results were mainly driven by the change from no TR at the baseline to mild TR at 12-month follow-up (baseline: no TR, 22 patients; mild TR, 10 patients; 12 months follow-up: no TR, 14 patients; mild TR, 17 patients; moderate TR, 1 patient). Importantly, none of the patients developed haemodynamically significant MR or TR in any of the arms at 12-month follow-up.

There were two device pocket infections in the LBBAP group requiring device extraction with contralateral implantation. Both infections occurred >12 months after the initial procedure. In the BiV group, one perforation of the atrial lead was managed with surgical intervention, and one dislocation of the LV lead which was repositioned 2 days after acknowledgement.

## Discussion

The CSP-SYNC randomized, non-inferiority trial aimed to compare LBBAP with conventional BiV pacing in patients with a Class I indication for CRT.

The main findings are as follows:

1. The change in LVEF after LBBAP was non-inferior to BiV pacing at the preplanned 6-month follow-up.
2. LBBAP provides a similar degree of LVESV reduction and QRS duration shortening compared to BiV pacing, as well as functional improvement in NYHA class and 6-min walk test distance.
3. While echocardiographic response was similar between both groups, normalization of ejection fraction (LVEF ≥50%) rates were significantly higher in the LBBAP group.
4. When observed over 12 months, the LBBAP group exhibited similar electrical and structural reverse remodelling.

### Left ventricular reverse remodelling

Standard BiV pacing is one of the most effective HF therapies, which leads to reverse LV remodelling and reduction in HF hospitalizations and all-cause mortality.^1–3^ The LVEF improvement in pivotal randomized BiV-CRT pacing trials that led to significant mortality reduction ranging from 6.9% at 18 months to 8% at 12 months.^2,3^ These improvement rates were surpassed by several observational studies comparing LBBAP and BiV pacing. In addition to greater improvement of LV function, significant QRS reduction, and reduction of HF-related hospitalizations were also shown with LBBAP.^8–12^

Recently, three randomized studies confirmed the resynchronization potential of LBBAP compared to BiV by demonstrating similar or greater improvement in LVEF.^13–15^ The results of our randomized trial align with these studies, indicating a similar degree of LV reverse remodelling and functional improvement with LBBAP compared to BiV pacing at the 6-month follow-up. In the randomized LBBP-RESYNC trial (Left Bundle Branch Pacing Versus Biventricular Pacing for Cardiac Resynchronization Therapy), which included 40 non-ischaemic HF patients with LBBB, intention-to-treat comparison showed a 5.6% higher LVEF improvement at 6 months after LBBAP than BiV pacing.^13^ In our study, a comparable mean intergroup difference of 5.6% LVEF improvement was detected, meeting the pre-specified non-inferiority margin. In contrast, randomized trials LEVEL-AT^14^ (Left Ventricular Activation Time Shortening With Conduction System Pacing vs. Biventricular Resynchronization Therapy) with 70 patients and the extended CONSYST-CRT^15^ (Conduction System Pacing vs. Biventricular Resynchronization Therapy in Systolic Dysfunction and Wide QRS) with 134 patients, showed similar improvement of LVEF compared to BiV pacing, but non-inferiority was not met at 6- and 12-month follow-up, respectively. The inclusion of a broader patient population in both trials that included individuals with permanent atrial fibrillation, non-LBBB QRS morphology (almost 16% of included patients had intraventricular conduction delay), and those undergoing device upgrades may have contributed to the discrepancy with our findings.

The analysis of time trends over a 12-month period revealed similar patterns of EF change and LVESV reduction in both groups. Notably, greater improvement in LVESV reduction in the LBBAP group was observed at 6 months. While our study was not designed to determine superiority or the impact on clinical events, these results, along with a trend indicating fewer HF hospitalizations in the LBBAP group compared to the BiV group (6.5% vs. 22.6%; *P* = 0.09), should be interpreted with caution. Nonetheless, the LV reverse remodelling observed during the short follow-up period suggests that LBBAP may have the potential to positively affect HF-related outcomes in patients with a Class I indication for CRT.

### Electrical remodelling

In our study, we observed a similar reduction of paced QRS duration in both groups at 6-month follow-up. While our findings align with the previous randomized trials,^13–15^ there is a discordance with several observational studies, which reported a greater reduction in QRS duration with LBBAP.^10–12^ Variations in the methods used for assessing QRS duration (using either the pacing stimulus or the initial QRS deflection as the starting point of measurement) and potential differences in optimizing BiV pacing devices may explain the discrepancies observed in our results. The observation could also be extracted from the recent CONSYST-CRT trial,^15^ where the difference in reduction of QRS between both groups was less prominent when QRS was measured from pacing spike compared to when measured from fast deflection point (mean difference of −1.0 ms vs. −4.0 ms, respectively). Since an isoelectric interval in all 12 leads at 100 mm/s is often present even with non-selective LBBAP, the QRS duration in our study was measured from the pacing stimulus as recommended by the CSP consensus.^19^ Nonetheless, a sustained pattern of electrical remodelling after LBBAP over 12 months might explain its favourable impact on structural reverse remodelling.

### Procedural characteristics and clinical implications

In the LBBP group, we observed a high success rate (96.8%) with a similar total procedure time to BiV pacing, but a shorter fluoroscopy duration. The complication rate was low. The success rate of LBBAP for CRT varies from 82.2% to 97%,^9,20^ seemingly higher than His bundle pacing.^6,7^ There are several potential reasons for the favourable procedural characteristics in our study. First, all LBBAP procedures were performed by two experienced operators, both exceeding the number of cases considered to mark the plateau of the learning curve in the MELOS study.^9^ Second, our study only included HF patients with Class I indication for CRT, excluding non-LBBB QRS morphology, which is a strong predictor of implantation failure and could result in higher cross-over rates.^15,21^ However, even LBBB QRS morphology on the surface ECG with Strauss criteria can be a result of distal conduction disease with intact Purkinje activation, which is not always amendable with LBBAP.^22^ Therefore, there is a need to establish additional criteria to identify patients who may benefit from this pacing technique alone or in combination with BiV pacing (left bundle-optimized CRT) to fully validate the benefits associated with LBBAP in the treatment of HF.^23^ Given that device-related valvular deterioration is associated with increased mortality,^24^ it is noteworthy that no patients in our study in either study arm developed haemodynamically significant MR or TR at the 12-month follow-up. These findings further support the feasibility of LBBAP for CRT without increasing the risk of lead-related valvular regurgitation. In clinical practice, effective LV electrical resynchronization from a single LBBAP lead could allow the use of a dual-chamber pacemaker as a valid treatment option for selected patients with HF and LBBB, which might improve the cost-effectiveness of CRT delivery, especially in resource-constrained settings. Moreover, the results of our randomized study provide additional evidence supporting the emerging paradigm that LBBAP may be considered as a potential first-line therapy for CRT indications, rather than being limited to a bail-out option when BiV pacing cannot be achieved. This finding should be interpreted in the context of current practice, where BiV pacing remains the preferred technique, as reflected in a recent European survey on the adoption of CSP.^25^

### Study limitations and strengths

The study has some limitations. First, the sample size to assess the non-inferiority of LBBAP compared to BiV pacing in LVEF improvement was based on 5% SD and a 2.5% non-inferiority margin, which might underestimate variability and overestimate effect size, potentially leading to an underpowered study that precludes definite conclusions regarding the long-term clinical efficacy. Second, a relatively short follow-up period was set for the primary endpoint. However, as part of a secondary analysis, we extended the echocardiographic follow-up to 12 months and observed non-significant changes in LV reverse remodelling in both modalities. This is consistent with findings from pivotal CRT-BiV randomized trials, which have also demonstrated limited additional echocardiographic improvement beyond 12 months of resynchronization therapy.^2,3^ Third, the present study was conducted in a single centre, which may limit the generalizability of procedural success rates and outcomes, particularly for the LBBAP modality. Finally, the interpretation of our results should be limited to HF patients with Class I indication for CRT meeting Strauss criteria for LBBB and should not be extrapolated to patients with different indications or to those with atrial fibrillation who represent a substantial proportion of the HF population and may also potentially benefit from LBBAP. Noteworthy, 32% of patients in the study had ischaemic cardiomyopathy, indicating that the observed benefits of LBBAP may extend beyond non-ischaemic aetiologies.

Despite these limitations, the study has several notable strengths. Adherence to guideline-directed medical therapy was excellent, with over 94% of patients receiving all four foundational HF medications, thereby reducing pharmacologic variability as a potential confounder. In addition, crossover between pacing modalities was minimal compared to contemporary randomized trials,^13–15^ with only one patient in the LBBAP group requiring conversion to standard BiV pacing and none in the opposite direction. This preserved the integrity of group allocation and supports the internal validity of the findings. Moreover, the quality of BiV implants in this study was high, as evidenced by QRS durations that were comparable to those achieved with LBBAP. Even so, LBBAP yielded comparable results with some superior findings, albeit exploratory, suggesting a potential advantage of the CSP pacing modality over BiV pacing. Taken together, these findings highlight the potential of LBBAP as an effective CRT strategy in appropriately selected patients. Larger multicenter randomized studies with longer follow-up and broader patient populations are needed to confirm these results and evaluate long-term clinical outcomes.

## Conclusions

In patients with a Class I indication for CRT, LBBAP was non-inferior to BiV pacing in improving LVEF and other echocardiographic and clinical endpoints at preplanned 6-month follow-up. Additionally, LBBAP provided a similar degree of structural and electrical reverse remodelling over 12 months and clinical outcomes during mid-term follow-up. These findings suggest that LBBAP could be a feasible alternative to BiV pacing and warrant further investigation in larger, multicenter trials.

## Supplementary Material

euaf192_Supplementary_Data

## Data Availability

The data underlying this article are available in the article and in its online supplementary material.
