# Supplementary material for: Conduction system pacing vs. biventricular pacing for cardiac resynchronization: the CSP-SYNC randomized single centre study
Source: Europace. 2025 Aug 23;27(9):euaf192. doi: 10.1093/europace/euaf192 (PMC13223735; doi:10.1093/europace/euaf192)
Supplement: euaf192_Supplementary_Data [file euaf192_supplementary_data.docx]

**Suplementary data**

The reported results for echocardiographic and clinical outcomes were corrected for multiple comparisons to support a more robust statistical interpretation. If correction for multiple analyses was not used, the intergroup difference for LVEF reached statistical significance for noninferiority also at 1 month (P = 0.021), which complements the significance at 6 and 12 months in the adjusted analysis. Furthermore, an uncorrected superiority analysis showed greater improvement in LVEF in the LBBAP group at 6 and 12 months (P = 0.006 and P = 0.004, respectively). In addition to the reported superiority for LVESV difference at 6 months, the uncorrected analysis also showed superiority of LBBAP at 12 months (P = 0.009). In addition to the previously reported noninferiority of QRS width differences at 6 and 12 months, further uncorrected analysis also showed noninferiority after 1 month (P = 0.021). Uncorrected analyses of NT-proBNP, 6-MWT, EQVAS score and change in NYHA class are provided in the supplementary data (Supplementary Table 1 and Table 2).

**Supplementary table 1:** Echocardiographic and electrocardiographic endpoints with unadjusted results of the longitudinal tests with non-inferiority P values and superiority P values in parentheses and 95% confidence intervals.

|  | Mean difference between groups  (95% CI)  **Baseline to 1 month** | P value | Mean difference between groups  (95% CI)  **Baseline to 6 months** | P value | Mean difference between groups  (95% CI)  **Baseline to 12 months** | P value |
| --- | --- | --- | --- | --- | --- | --- |
| ∆ LVEF (%) | 1.6 (-2.4, 5.6) | 0.021 (0.424) | 5.6 (1.6, 9.5) | < 0.001 (0.006) | 5.8 (1.8, 9.8) | < 0.001 (0.004) |
| ∆ LVESV (ml) | -8 (-28, 12) | 0.011 (0.417) | -24 (-44, -4) | < 0.001 (0.017) | -27 (-47, -7) | < 0.001 (0.009) |
| ∆ QRS (ms) | 2 (-8, 12) | 0.021 (0.733) | -1 (-11, 9) | 0.004 (0.787) | -2 (-12, 8) | 0.003 (0.708) |

LVESV= left ventricular end systolic volume, LVEF = left ventricular ejection fraction, QRS = duration of QRS complex from paced stimulus to end of QRS,

**Supplementary table 2:** Unadjusted results of differences in clinical endpoints after 6 months of therapy for non-longitudinal tests with P values for non-inferiority tests and P values for superiority tests in parentheses.

|  | Non-inferiority ∆ | P value non-inferiority | (P value superiority) |
| --- | --- | --- | --- |
| ∆ NT-proBNP | 100 ng/l | 0.365 | 0.889 |
| ∆ 6-MWT | 50 m | < 0.001 | 0.856 |
| ∆ EQ VAS score | 10 | 0.073 | 0.471 |
| ∆ NYHA functional class | 1 | 0.001 | 0.234 |

EQ VAS score = EuroQol Group visual analogue scale score, NYHA = New York Heart Association , NT-proBNP= N-Terminal Pro-B-Type Natriuretic Peptide, 6-MWT = 6-minute walk test

**Supplementary table 3:** Intraoperative measurements in the LBBAP arm.

| **Patient ID** | **QRS transition V1 – output** | **V6RWPT interval (ms)** | **V6RWPT** ≤**90ms** | **V6 - V1 interval (ms)** | **V6 - V1 >44 ms** | **Programmed stimulation** | **Paced QRS narrowing** | **Type of engagement** |
| --- | --- | --- | --- | --- | --- | --- | --- | --- |
| 1 | yes | 80 | yes | 52 | yes | NP | yes | S-LBBAP; SF |
| 2 | yes | 75 | yes | 45 | yes | yes | yes | S-LBBAP; SF |
| 3 | yes | 88 | yes | 46 | yes | yes | yes | NS-LBBAP; SF |
| 4 | yes | 80 | yes | 52 | yes | NP | yes | NS-LBBAP; AF |
| 5 | yes | 77 | yes | 38 | no | yes | yes | NS-LBBAP; AF |
| 6 | yes | 72 | yes | 47 | yes | NP | yes | S-LBBAP; truncus |
| 7 | yes | 80 | yes | 72 | yes | NP | yes | S-LBBAP; truncus |
| 8 | yes | 66 | yes | 40 | no | yes | yes | S-LBBAP; truncus |
| 9 | yes | 77 | yes | 44 | yes | NP | yes | S-LBBAP; AF |
| 10 | Crossover to BiV | / | / | / | / | / | / | HBP - no correction |
| 11 | yes | 63 | yes | 46 | yes | NP | yes | S-LBBAP; AF |
| 12 | yes | 66 | yes | 60 | yes | NP | yes | S-LBBAP; SF |
| 13 | yes | 89 | yes | 40 | no | yes | yes | S-LBBAP; SF |
| 14 | yes | 83 | yes | 69 | yes | NP | yes | S-LBBAP; AF |
| 15 | yes | 75 | yes | 40 | no | yes | yes | NS-LBBAP; SF |
| 16 | yes | 83 | yes | 40 | no | NP | yes | S-LBBAP; AF |
| 17 | no | 77 | yes | 60 | yes | NP | yes | LBBAP not clear |
| 18 | yes | 90 | no | 46 | yes | yes | yes | S-LBBAP; truncus |
| 19 | yes | 75 | yes | 45 | yes | NP | yes | S-LBBAP; PF |
| 20 | yes | 77 | yes | 40 | no | NP | yes | S-LBBAP; AF |
| 21 | yes | 88 | yes | 48 | yes | NP | yes | S-LBBAP; AF |
| 22 | yes | 80 | yes | 71 | yes | NP | yes | S-LBBAP; SF |
| 23 | yes | 80 | yes | 40 | yes | NP | yes | S-LBBAP; SF |
| 24 | yes | 94 | no | 52 | yes | NP | yes | S-LBBAP; PF |
| 25 | no | 92 | no | 40 | no | NP | yes | LBBAP not clear |
| 26 | yes | 83 | yes | 49 | yes | NP | yes | S-LBBAP; SF |
| 27 | yes | 66 | yes | 57 | yes | NP | yes | S-LBBAP; AF |
| 28 | yes | 83 | yes | 46 | yes | yes | yes | S-LBBAP; SF |
| 29 | yes | 80 | yes | 60 | yes | NP | yes | S-LBBAP; SF |
| 30 | yes | 80 | yes | 55 | yes | NP | yes | NS-LBBAP; PF |
| 31 GR | yes | 67 | yes | 55 | yes | NP | yes | NS-LBBAP; AF |

LBBAP = left bundle branch area pacing, NS = nonselective, S = selective, PF = posterior fascicle, AF = anterior fascicle, SF = septal fascicle, ms = miliseconds, NP = not performed.
